# Supplementary material for: Quality of newborn care and associated factors: An analysis of the 2022 Kenya demographic and health survey
Source: PLOS Glob Public Health. 2024 Nov 13;4(11):e0003677. doi: 10.1371/journal.pgph.0003677 (PMC11560034; doi:10.1371/journal.pgph.0003677)
Supplement: Attachment — Submitted filename: PGPH-D-24-01885.pdf [file pgph.0003677.s001.pdf]

Review: Quality of newborn care and associated factors: An analysis of the 2022 Kenya demographic and health survey.

"Quality of newborn care and associated factors: An analysis of the 2022 Kenya demographic and health survey." by Asiimwe JB et al., a manuscript on a very important aspect regarding newborn care – to identify the factors associated with the quality of newborn care in Kenya.

The world has made substantial progress in child survival since 1990. However, the decline in neonatal mortality has been slower than that of post-neonatal under-5 mortality. Plans to improve newborn survival should be built on a strong foundation of essential newborn care and align with Every Newborn Action Plan (ENAP) and Ending Preventable Maternal Mortality (EPMM) targets on antenatal care, postnatal care, skilled health personnel and emergency obstetric and newborn care.

The authors have tried to find out the condition of the factors that influence the quality of newborn care in Kenya using data from nationally representative survey, Kenya Demographic and Health Survey (KDHS) 2022. They have several recommendations from the findings for better ensuring the accessibility and quality of neonatal health services. I believe identifying the gaps in quality of newborn care through this publication and taking appropriate measures will help to improve neonatal care in in Kenya and elsewhere in developing countries.

Although it is well written, I have few observations, as mentioned below, that need to be addressed before publication.

Line 66: 'a third of the neonates' is fine, '(1 in 3)' probably is not necessary.

Line 100: Here emphasis has been given on 'delayed initiation of breast feeding'. Early initiation of breastfeeding, within one hour of birth, protects the newborn from acquiring infection and reduces newborn mortality. It seems to me that his sentence does not well fit here, also does not go with the next sentence. It could be an important finding if data was there about the time of initiation of breast feeding.

Line 119: Here mentioned 'some studies', but reference is given for one study [31].

Line 210: 'Most participants gave birth while they were under 24 years old (87.1%)'. It is the information about the age at first birth (Table 1), not of the delivery that was surveyed, this should be clarified.

Line 216: About 1 in 10 women will have a miscarriage over a lifetime, according to a series of articles in The Lancet. Is 23% fewer pregnancy loss? Does this figure match with data given in Table 3? Probably there is many missing data, need comments on that.

Table 3: I have confusion about the numbers of cases, I cannot match them. What is the denominator?

Line 303-35: Babies born by ceasarian section might have higher incidences of neonatal complications and mortality probably due the indication that necessitated the ceasarian section. There is no reason that such babies are prone to adverse conditions like 'concomitant diarrhea and acute respiratory infections'. The first publication in the reference mentioned babies aged less than 6 months and the second publication is on a special situation.

Line 355: 'Abbreviations' are probably not required separately. The rule is to spell out the full term in text at its first mention, its abbreviation in parenthesis and the abbreviation from then on.

Gestational age (term or preterm) and birth weight (AGA or LBW) is a is major determinant of neonatal morbidity and mortality. I am not sure whether this information was available for analysis.
