# Supplementary material for: Quality of newborn care and associated factors: An analysis of the 2022 Kenya demographic and health survey
Source: PLOS Glob Public Health. 2024 Nov 13;4(11):e0003677. doi: 10.1371/journal.pgph.0003677 (PMC11560034; doi:10.1371/journal.pgph.0003677)
Supplement: Attachment — Submitted filename: Response to Reviewers.docx [file pgph.0003677.s002.docx]

**Manuscript title:** Quality of newborn care and associated factors: An analysis of the 2022

Kenya demographic and health survey

| **#** | **Reviewer comment** | **Response** | **Page/Line number** |
| --- | --- | --- | --- |
| 1 | 1. Please review your reference list to ensure that it is complete and correct. If you have cited papers that have been retracted, please include the rationale for doing so in the manuscript text, or remove these references and replace them with relevant current references. Any changes to the reference list should be mentioned in the rebuttal letter that accompanies your revised manuscript. If you need to cite a retracted article, indicate the article’s retracted status in the References list and also include a citation and full reference for the retraction notice. | All papers included in the manuscript are cited. All doi numbers have been added  Changed references are added.  No retracted articles are cited.  All removed references have mentioned below | **Pg 20-24** |
|  | **Reviewer one** | | |
| 2 | “Quality of newborn care and associated factors: An analysis of the 2022 Kenya  demographic and health survey.” by Asiimwe JB et al., a manuscript on a very important  aspect regarding newborn care – to identify the factors associated with the quality of  newborn care in Kenya.  The world has made substantial progress in child survival since 1990. However, the  decline in neonatal mortality has been slower than that of post-neonatal under-5  mortality. Plans to improve newborn survival should be built on a strong foundation of  essential newborn care and align with Every Newborn Action Plan (ENAP) and Ending  Preventable Maternal Mortality (EPMM) targets on antenatal care, postnatal care, skilled health personnel and emergency obstetric and newborn care.  The authors have tried to find out the condition of the factors that influence the quality of newborn care in Kenya using data from nationally representative survey¸ Kenya  Demographic and Health Survey (KDHS) 2022. They have several recommendations from the findings for better ensuring the accessibility and quality of neonatal health services. I believe identifying the gaps in quality of newborn care through this publication and taking appropriate measures will help to improve neonatal care in in Kenya and elsewhere in developing countries.  **Although it is well written, I have few observations, as mentioned below, that need to be addressed before publication.** | Thank you very much for your compliments and constructive feedback that has helped us to improve our manuscript. We have revised the paper following your suggestions. | N/A |
|  | Line 66: ‘a third of the neonates’ is fine, ‘(1 in 3)’ probably is not necessary. | This has been revised as suggested. | Line 57 |
| 3 | Line 100: Here emphasis has been given on ‘delayed initiation of breast feeding’. Early  initiation of breastfeeding, within one hour of birth, protects the newborn from acquiring  infection and reduces newborn mortality. It seems to me that his sentence does not well  fit here, also does not go with the next sentence. It could be an important finding if data was there about the time of initiation of breastfeeding. | This sentence has been revised to better flow with the next statement. | Line 90-91 |
|  | Line 119: Here mentioned ‘some studies’, but reference is given for one study [31]. | Another reference has been added <https://doi.org/10.1136/bmjgh-2020-004475> | Pg 4, LN 112 |
| 4 | Line 210: ‘Most participants gave birth while they were under 24 years old (87.1%)'. It is  the information about the age at first birth (Table 1), not of the delivery that was  surveyed, this should be clarified. | We have clarified this statement “Most participants first gave birth while they were under 24 years old (87.1%)” | Pg 7, LN 199 |
| 5 | Line 216: About 1 in 10 women will have a miscarriage over a lifetime, according to a  series of articles in The Lancet. Is 23% fewer pregnancy loss? Does this figure match with  data given in Table 3? Probably there is many missing data, need comments on that.  Table 3: I have confusion about the numbers of cases, I cannot match them. What is the  denominator? | Thank you very much for the critical comment. Table 1 included all participants that responded to the interview on newborn care. Table 2 included participants who responded to specific questions related to newborn care (individual care service received). The responses varied depending on the newborn service received. Some were more received than others, which is why they vary. Table 3 included all participants who responded to all questions related to newborn care. This means if a participant skipped or did not respond to some questions related to the quality of newborn care (some missing figures), and others who did not respond at all to all questions (all missing figures), they were not included in the final analysis shown in Table 3. Therefore, these were not included in the analysis (We included in the analysis all participants who responded to all the questions related to newborn care as shown in Table 3. This is the reason why Table 1, has a large number of participants followed by Tables 2 and 3. **Disclaimers have been added to the table legends**. In addition, the issue of the missing data has been added to the limitation section of this study. it now reads: “*Finally, due to the large number of missing data points, we included fewer participants in univariate and multivariate logistic regression analyses.”* | Pg 9 LN212-213;  Pg 11 LN 226-228;  Pg 15, 251-253;  Pg 18, LN 337-338 |
| 6 | Line 303-35: Babies born by ceasarian section might have higher incidences of neonatal  complications and mortality probably due the indication that necessitated the ceasarian  section. There is no reason that such babies are prone to adverse conditions like  ‘concomitant diarrhea and acute respiratory infections’. The first publication in the  reference mentioned babies aged less than 6 months and the second publication is on a  special situation. | Thank you very much for your comment. Your suggestion has been put into account and cited and the old assertions and their citations have been eliminated to match the new changes. | Pg 16 LN 303-304 |
| 7 | Line 355: ‘Abbreviations’ are probably not required separately. The rule is to spell out the  full term in text at its first mention, its abbreviation in parenthesis and the abbreviation from then on. | Thank you very much for your suggestion. The Abbreviations table has been deleted as suggested. | Pg 18 |
| 8 | Gestational age (term or preterm) and birth weight (AGA or LBW) is a is major determinant of neonatal morbidity and mortality. I am not sure whether this information was available for analysis. | Thank you very much for your suggestion. Unfortunately, the suggested variables were not available in the datasets. |  |
|  | **Reviewer two** | | |
| 9 | Many thanks for your work on this critical subject matter - Quality of newborn care and associated factors.  Generally the method using a 2-stage stratified sampling across 8 regions in Kenya is a valid and interesting topic. However, upon delving into this manuscript I found a number of improvements that would make it more convincing and stronger.  Generally, the construction of "Quality" care has been fairly difficult to pin down - I wonder why the WHO framework (2016) for quality of newborn care was not relied upon to define the provision of quality care. Instead of or in addition to REF3 for recommendations for "a positive childbirth experience." | The WHO framework definition has been added, cited and it reads““*According to World Health Organization (WHO) the quality of newborn care is the extent to which newborn health care services meet the desired health outcomes of newborns and must be provided in a safe, timely, and appropriate manner while putting into the needs (or preferences) of newborns and their families*.” The old reference (1) was eliminated. | Pg 3 LN 68-71 |
| 10 | OR another more sensible proximal to distal modality (bodily, maternal, delivery, after care, parental, SES/demographic) to list the risk factors impinging on newborn quality care provision. Regardless, the risks are presented here and compared with other previous work in the field so it just begs for a more hard definition. | Thanks a lot for the suggestion on how we can organize our predictors. Nonetheless, we did organize our predictors based on conventional literature reviewed. We did not want to differ much from what other studies suggested. |  |
| 11 | Also, please remember that many people are not aware with which regions of Kenya are marginalized or not (ln 69). | We have specified the regions where the quality of newborn care was found to be low. The statement is changed from marginalized to areas where the quality of newborn care was found to be low. It now reads” *from certain regions of Kenya where the quality of newborn care was found to be low*” | Pg 2 LN 60 |
| 12 | There was another statistical oddity that I thought undermined the paper's credibility and should be revised. In table 1 Religions are listed as Christian, Muslim and Others. I see that the regression was performed using "Other" as the nominal or base and then concluding that both Christian and Muslim religions seem to be protective and encourage quality of care. Where it would be more convincing to me that the "other" religions were in fact the marginalised groups (ln69). This is exacerbated by the number of Others (209 1.8%) and it is generally not good practice to use a small and under-representative group as the base to a regression is not suitable. | Thank you very much for your constructive feedback. The percentage for the other faith is indeed a little bit low compared with Christians and Muslims. Since there were two major religions in our initial analysis the base was either Muslim or Christian but no statistical significance was found. However, when compared with other faiths we found a statistically significant difference, and that is what we report in our study. We recognize that this may have its weaknesses but given that the binary comparison did not violate the cell chi-square assumptions the finding may still be credible. |  |
| 13 | I was quite thrown by the caesarian findings as well, these must take place in a facility, and it was odd to see that many shortfalls related to quality happen there, including failing to weigh the baby? | Thanks for the comments. But that is what we found in the analysis. |  |
| 14 | You mention that dummy variables were constructed but do not specify which or how these composite variables are created or compared. it is assumed that functionally this would happen in line, so either define these or omit the dummy vars (178) | Thanks a lot for your suggestions. Because multiple variables were collapsed into new variables with better meaning, we could not provide specific examples. *We have reconsidered to omit the term “dummy variables.”* | Pg 6 LN 168 |
|  | Your introduction seems to list a number of risks and such from various places, but does not clearly assert that you will be defining your risks as X based on Y or Y+Z. Thus the reader is left guessing until the line 147 Dependent variables section or Table 1 essentially. Consider a complete revision and tightening up. | Thanks a lot for your suggestions. The operational definition of quality of newborn care as used in this study has been added to the introduction as well as how it was compared with various parental, and obstetric characteristics. Thus, we have restated our objective as;  *Therefore, this study aimed to identify the parental, health facility, obstetric, and child-related factors associated with the quality of newborn care (ie., receiving all the newborn care services) as reported by the mothers using nationally representative data from Kenya.* | Pg 4 LN 112-115 |
| 15 | In fact, the conclusions seem quite foregone, that quality risk comes from facilities and parental factors. | Thanks a lot for the suggestion. The following statement has been added to the conclusions section “*This study also seems to suggest that facility-related and parental social factors are associated with receiving quality newborn care.* | Pg 2 LN 58 |
| 16 | Here are some additional comments to crosscheck and address. 1. Stata version seems to be out of date (ln 44) 2. ln 55 - add "based on the above definitions" to the end of the sentence to clarify  3. 183 typo Odd"s" ratio.  4. ln 79 - Very awkward wording revise  5. ln 80 - change in to "to"  6. ln 81 - change "thus, reducing" to "and can reduce"  7. ln 117 - consider change "monitor the quality" to "invest in monitoring the quality"  8. ln 148 - consider revision to clarify method of aggregate variable creation here. is it a binary in case of total quality 1/0 or some other cut-off - may also consider nodding to other risk scoring efforts somewhere.  9. ln 160 - you list 4 categories but say 3.  10. ln 213 - 30mins - needs space  11. ln 228 remove "babies"  12. ln 260 - respective to what, need a pre-list  13. ln 271 - the title of ref 48 does not specify where  14. ln 291 - reference is from Nepal, perhaps not apt.  15. ln 340 - informal voice – remove | Thanks a lot for the constructive feedback. The version of spss has been updated to v29 in the abstract and the methods  The term odds ratio has been corrected  The sentence on line 79 has been replaced with the correct definition by WHO.  Ln 80, “in” has been changed *“to assisting”*  The sentence has been changed to *“and can reduce neonatal..”*  The sentence “invest in monitoring the quality” has been added.  Since all the variables were ranked as 0 (no) or 1 (yes), the yes to all variables was ranked as having received quality newborn care and vice versa. This is clarified at the end of this section (binary aggregation)  The number of categories has been corrected.  The space has been added it now reads“ 30 minutes)  The word “babies” has been removed.  The word “respectively has been removed.  It is true that the study country is not included in the title of that article (see: . doi: 10.3390/children10071141)  It's true that the reference is from Kenya. This has been corrected  The sentence has been edited and the informal voice removed | Pg 2 LN 40  Pg 6 LN174  Pg 6 LN 173  Pg3LN68-71  Pg 3 LN 71  Pg 2 LN 72  Pg 4 LN 107  Pg 5LN 147  Pg 5 LN 150  Pg 7LN 202  Pg10 LN222  Pg15 LN258  Reference 50  Pg16 LN281  Pg18 LN343 |
| **17** | **Editor Comments:**  The submitted manuscript titled "Quality of Newborn Care and Associated Factors: An Analysis of the 2022 Kenya Demographic and Health Survey" explores the quality of newborn care in Kenya. The findings of this study is particularly valuable for many LMICs, where essential newborn care is often difficult to access. The paper identifies several barriers to improving newborn care in Kenya, which may also be relevant in other regions with high neonatal mortality rates. However, the reviewers have noted some weaknesses/limitations in the manuscript that must be addressed adequately before it can be considered for publication. | Thanks for the compliment, the paper has been revised according to the reviewer's comments. |  |
