# Supplementary material for: Quality of newborn care and associated factors: An analysis of the 2022 Kenya demographic and health survey
Source: PLOS Glob Public Health. 2024 Nov 13;4(11):e0003677. doi: 10.1371/journal.pgph.0003677 (PMC11560034; doi:10.1371/journal.pgph.0003677)
Supplement: Attachment — Submitted filename: Response to reviewer comments.docx [file pgph.0003677.s003.docx]

| **#** | **Reviewer comment** | **Response** | **Page/Line number** |
| --- | --- | --- | --- |
| Previous comment | There was another statistical oddity that I thought undermined the paper's credibility and should be revised. In table 1 Religions are listed as Christian, Muslim and Others. I see that the regression was performed using "Other" as the nominal or base and then concluding that both Christian and Muslim religions seem to be protective and encourage quality of care. Where it would be more convincing to me that the "other" religions were in fact the marginalised groups (ln69). This is exacerbated by the number of Others (209 1.8%) and it is generally not good practice to use a small and under-representative group as the base to a regression is not suitable. | Thank you very much for your advice. We changed the comparison group or base to Christian which had the largest number of participants. The change in findings have been reflected in table 3, results section, discussion and abstract. | Pg 2, LN 52-54  Pg 11 LN239-241  Pg 12  Pg 15 LN 249  Pg 16 LN 287-289 |
| Current revision #1 | The revised version of the manuscript addresses most of the concern raised by the reviewers. However, we suggest reconsidering the concern raised by the second reviewer regarding the use of 'other religion' as the reference category in Table 3 to estimate the impact of religion on receiving quality newborn care. Due to the smaller sample size in the other religion category, this choice may undermine the credibility of the paper, and the interpretation should be revised accordingly. | Thank you very much for your advice. We changed the comparison group or base to Christian which had the largest number of participants. The change in findings have been reflected in table 3, results section, discussion and abstract. | Pg 2, LN 52-54  Pg 11 LN239-241  Pg 12  Pg 15 LN 249  Pg 16 LN 287-289 |
| #2 | Additionally, as pointed out by Reviewer 1, in Table 3, please mention the total number of participants analyzed to ensure readers can understand how the percentages were calculated. | Thank you very much for the advice. The total number of participants analyzed (N=4600) has been added to Table 3 | Pg 11 |
