# Supplementary material for: Quality of newborn care and associated factors: An analysis of the 2022 Kenya demographic and health survey
Source: PLOS Glob Public Health. 2024 Nov 13;4(11):e0003677. doi: 10.1371/journal.pgph.0003677 (PMC11560034; doi:10.1371/journal.pgph.0003677)
Supplement: Attachment — Submitted filename: Response to reviewer comments.docx [file pgph.0003677.s004.docx]

| **#** | **Reviewer comment** | **Response** | **Page/Line number** |
| --- | --- | --- | --- |
| Previous comment | Thank you for sharing the revised version of the manuscript. It adequately addresses the reviewers' concerns. However, in Table 3, the percentage calculations appear incorrect. It seems the authors calculated the percentage for each cell based on the total number of respondents. Additionally, some categories (e.g., mother's age) do not sum to 100%. Ideally, the percentages for each row (Yes and No) should add up to 100%. Please revisit the percentage calculations and correct if there is any errors. | Thank you very much for your comment and oversight. We have recalculated the percentages in Table 3 to make sure they add up to 100%. | Pg 11-15 |
